# Healthcare resource utilization analysis in newly diagnosed mantle cell lymphoma: resource alleviation with adoption of the TRIANGLE ibrutinib regimen without autologous stem cell transplantation

**DOI:** 10.1007/s00277-026-07139-1

**Published:** 2026-07-16

**Authors:** Jonas Wißkirchen, Nora S. Rogmann, Michael Greiling, Frederic Ries, Anke Ohler, Georg Hess, Julia Osygus

**Affiliations:** 1 Department of Hematology and Medical Oncology, Medical School of the Johannes Gutenberg-University, Mainz, Germany; 2 Institute for Workflow-Management in Health Care (IWiG), European University of Applied Sciences, Cologne, Germany; 3 Institute for Workflow-Management in Health Care (IWiG), Telgte, Germany

**Keywords:** Cost, HCRU, HDCT and ASCT, Mantle cell lymphoma, TRIANGLE ibrutinib regimen without ASCT

## Abstract

For younger, fit patients with mantle cell lymphoma (MCL), first-line therapy includes high-dose chemotherapy (HDCT) and autologous stem cell transplantation (ASCT). HDCT-ASCT demands considerable healthcare resources and costs and causes acute and long-term complications. The phase 3 TRIANGLE trial found clinically beneficial improvements in overall survival and safety when HDCT-ASCT was replaced with an ibrutinib regimen without ASCT. This study aimed to develop a comprehensive ASCT treatment clinical pathway model for patients newly diagnosed with MCL and assess the impact on hospitalization duration, healthcare resources, and cost. The clinical pathway was modeled using ClipMed^®^PPM software-based process analysis. Data were sourced from the IWiG^®^ reference model for quantitative activity-based health economic evaluation and validated by clinical experts alongside standard operating procedures. Cost inputs were obtained using reimbursement sources and tariff publications in Germany. The total clinical pathway, from ASCT-eligibility assessments, induction therapy, HDCT-ASCT consolidation, and up to 100 days post-ASCT, covered: 49 patient-clinician interaction days; 346 patient services (inpatient and outpatient settings); 1653 processes. HDCT-ASCT involved 23 days’ hospitalization. Healthcare professionals’ time associated with apheresis and HDCT-ASCT, which could be spared with the TRIANGLE ibrutinib regimen without ASCT, totaled 214 h. Costs associated with the total clinical pathway, excluding those associated with severe adverse events, other comorbidities, and long-term complications, amounted to €50,815 per patient. For newly diagnosed ASCT-eligible patients with MCL, HDCT-ASCT is associated with at least 23 hospitalization days, considerable costs, and substantial healthcare resources – assets that could be redirected with adoption of the TRIANGLE ibrutinib regimen without ASCT.

**Supplementary Information:**

The online version contains supplementary material available at 10.1007/s00277-026-07139-1.

## Introduction

Mantle cell lymphoma (MCL) represents approximately 5% of all non-Hodgkin lymphoma cases [1], with an overall incidence of 0.8 to 1.3 cases per 100,000 people across Europe [2–6]. MCL is an aggressive and incurable lymphoma associated with poor prognosis and short survival compared with other lymphoid cancer types [7, 8].

For younger, fit patients with MCL who have a good performance status, dose-intensified chemoimmunotherapy (CIT; such as rituximab plus cyclophosphamide, doxorubicin, vincristine, and prednisone [R-CHOP] alternating with rituximab plus dexamethasone, cytarabine, cisplatin and prednisone [R-DHAP]), followed by consolidation with high-dose chemotherapy (HDCT) and autologous stem cell transplantation (ASCT) with rituximab maintenance until recently represented the principle standard of care for first-line management in Europe [9, 10]. While these therapies can improve outcomes for patients with MCL, they are associated with a high incidence of relapse, treatment-related mortality, and long-term complications [11–13]. HDCT and ASCT treatment phases can contribute to early death, with a treatment-related mortality rate of 2% to 7% reported at 100 days post-ASCT [14–16]. HDCT and ASCT can also add significant burden on patients due to adverse events (AEs), hospitalization, and isolation during this period [1, 12, 17]. Among patients undergoing HDCT and ASCT, median hospital duration is 30 days (range, 18–51 days) [17], and approximately 3% to 5% of patients are later admitted to the intensive care unit (ICU) due to complications [13, 17], often sepsis [18]. Post-ASCT procedure, most (98%) survivors experience at least one moderate or more severe late effect (≥ 8 years posttreatment), and approximately half experience severe or life-threatening late effects [12]. In addition to the patient burden, the ASCT procedure incurs significant costs to healthcare bodies, with average costs of €107,457 per patient reported in Germany for aggressive lymphomas [19].

The randomized phase 3 TRIANGLE (NCT02858258) clinical trial of the European Mantle Cell Lymphoma Network investigated whether the addition of ibrutinib to induction and maintenance therapy resulted in superior clinical outcomes compared with the pretrial CIT standard with HDCT-ASCT, or ibrutinib plus CIT without HDCT-ASCT [20, 21]. The addition of ibrutinib resulted in superior failure-free survival (FFS) [20, 21]. However, the Kaplan-Meier plots for both ibrutinib-containing cohorts showed overlapping FFS [20, 21]. On the other hand, the observed toxicity during the ASCT and maintenance phase in the ASCT-cohorts seem to favor an ASCT-free approach [20, 21]. At a median 54.9 months of follow-up, treatment with ibrutinib plus CIT demonstrated superior 4-year FFS while omitting the burden of HDCT-ASCT (81% vs. 70%; hazard ratio [HR] 0.69; 2-sided 96.67% confidence interval [CI] 0.45–0.97; 2-sided *P* = 0.021) [22]. Additionally, ibrutinib plus CIT provided significantly longer 4-year overall survival (OS) versus HDCT-ASCT with CIT (90% vs. 81%; HR 0.57; 95% CI 0.36–0.90; 2-sided *P* = 0.0019) [22]. No meaningful differences between ibrutinib plus HDCT-ASCT vs. ibrutinib-only were observed for FFS (HR [1-sided 98.33% CI] 0.86 [0-1.27], 1-sided *P* = 0.21) [22]. The ibrutinib regimen (without HDCT-ASCT) from TRIANGLE is now recommended by the European Society for Medical Oncology, as well as several regional treatment guidelines [10, 23–27], and has received an indication extension approval from the European Medicines Agency as frontline therapy for patients with MCL who would be eligible for ASCT [28, 29].

While the burden of HDCT-ASCT is well understood, and the clinical value of replacing HDCT-ASCT with ibrutinib oral treatment has been demonstrated, it is unknown how this change in standard of care impacts healthcare resource utilization (HCRU). Considering the current burden of HDCT-ASCT therapy, and in light of the efficacy data reported from the TRIANGLE trial, this study aimed to determine hospitalization duration, HCRU, and direct costs associated with the HDCT-ASCT phase of the clinical pathway for eligible patients newly diagnosed with MCL – resources that could be redirected with the adoption of the TRIANGLE ibrutinib regimen.

## Methods

### Objectives

The primary objective was to develop a comprehensive model for the total ASCT therapy clinical pathway, encompassing detailed mapping of all pertinent processes for patients with MCL, and quantify healthcare resource utilization. For the purpose of this analysis, the term ‘process’ refers to subprocesses (i.e., clearly delineated, standardized, and repeatable units within the clinical pathway; each subprocess consists of a bundle of related activities triggered by specific inputs, performed by defined healthcare professionals, characterized by a measurable duration, and leading to a specific output or outcome) unless otherwise specified. The total clinical pathway started from the tumor board assessment (135 days prior to transplantation) and covered induction therapy (six 21-day cycles, alternating R-CHOP with R-DHAP) [21], apheresis, HDCT, ASCT, posttransplant hospitalization and monitoring (with patient discharge typically occurring 15 days posttransplantation), and long-term follow-up until 100 days post-ASCT. The secondary objective was to evaluate the costs associated with the ASCT therapy.

### Data source

The data for this study were derived from 5 sources: (1) IWiG (Institut für Workflow-Management im Gesundheitswesen) reference model for quantitative activity-based health economic assessment [30]; (2) TRIANGLE study protocol to define MCL disease-specific treatment phases and processes; (3) hospital standard operating procedures (SOPs); (4) clinical experts from the Department of Hematology and Medical Oncology, Medical School of the Johannes Gutenberg-University, Mainz, Germany; and (5) standard reimbursement sources and tariffs publications in Germany.

### Data analysis

#### Software-based procedural health economic analysis

To establish the ASCT clinical pathway for patients newly diagnosed with MCL, a ClipMed PPM software-based procedural health economic analysis (“Softwarebasierte Prozessuale Gesundheitsökonomische Analyse” [SPGA]) was conducted [31]. Proprietary ClipMedPPM software was used to model the clinical process flow for all procedures, spanning the first to the last doctor-patient contact, in both inpatient and outpatient settings, referred to hereafter as the total clinical pathway. The key milestones of the SPGA methodology are:

1. Determination of core competencies and clinical pathway, including: Identification of a homogeneous patient group with a specific medical condition, which could be assigned to a specific diagnosis-related group (DRG) with a comparable diagnosis, procedures (diagnostics, therapy), and treatment duration; and development of the clinical pathway including all services, such as diagnostics, therapies, supportive services, and other ancillary services based on clinical SOPs and guidelines with the assistance of the healthcare professionals (HCPs) responsible (e.g., physician, nurses, specialized functions).
2. Premodeling: Based on the clinical pathway, modeling of a “draft” process flow, comprising of all sub-processes from admission to discharge, using the ClipMed PPM software [30].
3. Main modeling – model validation: resources type and unit utilization collection: Adaptation of the premodeled clinical pathway and process flow; input and data collection, such as process duration, responsibilities, and probability of execution; data validation through structured individual and/or workshop settings discussions with physician and nursing staff.
4. Data processing and allocation: Processing of standard reimbursement sources and tariffs data [32], calculation and allocation of cost, using ClipMed PPM software [30].
5. Quality control and validation: Quality assurance for completeness, relevance, plausibility, and consistency.

This analysis included comprehensive modeling of treatment components and processes, assessments of process duration (measured in minutes), the roles and responsibilities of personnel involved, and the probabilities of successful execution at each stage.

The total clinical pathway was defined for a homogeneous patient group with a specific medical condition, which could be assigned to a specific DRG with a comparable diagnosis, procedures, and treatment duration. This model deliberately excluded transfers to the ICU, management of severe AEs, and other comorbidities unrelated to MCL, representing a typical patient on a hematology ward. With the noted exclusions, the study aimed to represent HCRU for a homogeneous patient population, demonstrating a standardized, clinically significant, and frequently provided form of care.

### Data validity

Data validity refers to both the application of the underlying model across multiple projects and the study-specific expert-based validation process.

#### Model

The ClipMed PPM software is applied within the framework of a process-based health economic methodology (SPGA [31]), which is independent of the software tool itself. The software serves as a modeling and calculation environment for structuring, visualizing, and quantifying clinical pathways and associated resource utilization. The underlying process reference model has been developed and continuously refined based on more than 300 clinically established pathways and validated in over 150 projects across different healthcare settings [30]. This extensive application base supports the methodological robustness and transferability of the approach. Model validity is ensured through a multi-step validation process, including structured expert input, iterative model refinement, and cross-validation against comparable clinical pathways. Process structures, time estimates, and resource allocations are systematically reviewed for completeness, plausibility, and consistency. Consequently, the reliability of the model outputs is primarily determined by the standardized modeling framework, the use of validated reference structures, and the integration of expert knowledge, rather than by the software tool alone.

#### Study-specific expert-based validation process

Expert validation was conducted using a structured, multi-step approach. First, initial model assumptions and process structures were derived based on the process reference model, existing clinical SOPs, and relevant clinical guidelines. These preliminary structures were subsequently reviewed and refined through iterative consultations with clinical experts from relevant disciplines, including hematology, oncology, nursing, and functional services. Data elicitation was performed through structured individual interviews and moderated workshops, in which process steps, time requirements, responsibilities, and resource allocations were systematically assessed and aligned. Multiple rounds of iteration were used to progressively refine the model and ensure consistency with real-world clinical practice. In cases of differing expert opinions, a consensus-based approach was applied. Divergent perspectives were discussed and reconciled by considering clinical guidelines, SOPs, and practical experience. Where uncertainty remained, decisions were guided by comparison with analogous processes from the underlying reference model. The data inputs used in the model are based on a combination of clinical guidelines, SOPs, standardized reimbursement and cost data sources, as well as validated reference processes. This approach ensures a methodologically robust and transparent derivation of model parameters.

### Cost analysis

The resource cost was calculated based on costs reported in German national reimbursement sources and tariff publications, such as the Institute for the Hospital Remuneration System calculation determining public DRG-reimbursement system in Germany (Institut für das Entgeltsystem im Krankenhaus GmbH [InEK]) [32].

#### Cost categories (standardized model based on publicly available data)

For the present health economic analysis, institution-specific controlling data were not utilized. Instead, cost estimation was based on standardized, publicly accessible sources such as collective labor agreements and InEK cost calculation data for the respective German DRG. This approach enabled a comparable, generalizable, and methodologically transparent representation of cost structures that extend beyond individual hospitals.

Personnel costs were calculated using average wage levels derived from applicable tariff agreements for typical healthcare professions, including doctors (e.g., hematology), nursing staff, and personnel from functional departments (e.g., radiology, cardiology, neurology), as well as support services. As such, the model does not reflect institution-specific staffing structures but rather provides a robust cross-sectional representation of typical healthcare delivery settings.

Direct material costs encompass commonly administered medications and medical interventions, such as cytostatic agents and supportive comedications, and are derived from national average values according to the InEK cost matrix. An exception applies to medication costs (e.g., rituximab). The general medication costs from the InEK matrix were reduced accordingly to account for the fact that the averaged InEK data include costs reimbursed separately through additional payments (Zusatzentgelte [ZE]). In the scope of this analysis, only the supplementary payments (ZE) and comparable medication costs from a representative case with a similar clinical treatment course were considered.

Additional costs for infrastructure, laboratory services, imaging, and overhead were modeled with a distinction between medical and nonmedical infrastructure. Considered categories included diagnostic units (e.g., laboratories), therapeutic services (e.g., ICUs), patient admission areas, and administrative or service departments (e.g., management, information technology, cleaning, and security).

This standardized modeling approach yields a representative and transferable cost scenario that reflects the economic relevance of the care pathway in a realistic and broadly applicable manner.

#### Inpatient setting

R-DHAP chemotherapy cycles, apheresis, and HDCT-ASCT all occur in the inpatient setting and are covered by the DRG codes R61H, A42C, and A15C, respectively. Additional payments (ZE) for relevant medications are included in the DRG reimbursement values and are allocated costs equating to material costs associated with each respective process. Due to the cost/case structure of the InEK matrix, the ZE-relevant medication costs are underrepresented based on the averaged data and do not realistically match the assumed ZE compensation. Additionally, double counting should be avoided; therefore, the related drug costs were deducted proportionally from the InEK cost matrix for the purpose of this analysis.

It was assumed that 85% of the drug costs in DRG R61H and DRG A15C were relevant to ZE and, therefore, 15% of the drug costs in the InEK matrix cost category “group 4 medications” (“4. Arzneimittel,” KAG 4) were distributed over the surcharges as standard medications. For the DRG A42C, it was assumed that 77.5% of the drug costs were reimbursed through additional fees. Correspondingly, 22.5% of the drug costs shown in the InEK cost matrix were included as surcharges in the cost analysis. These values are based on established calculation approaches for separating ZE-relevant medications from those integrated into the DRG. The specific proportion represents a model-based assumption derived from typical DRG and additional payment cost structures and should not be interpreted as a universally fixed parameter.

#### Outpatient setting

The remuneration for outpatient aftercare services is estimated based on the presumed cost flat rate GOP 86,512 of the oncology agreement (indicated as €250/quarter, according to regional Statutory Health Insurance agreement), supplemented by separately billable services such as laboratory diagnostics and imaging. Participation in an interdisciplinary tumor conference is covered by the flat rate in this case and is not considered separately. Duration (in quarters) was assumed in the cost calculation considering the typical total clinical pathway time.

The study was conducted from October 2024 to May 2025.

## Results

### Mapping procedure steps and processes

The total clinical pathway from the initial assessment of ASCT eligibility to 100 days post–HDCT-ASCT covered 49 patient-clinician interaction days, both in the inpatient and outpatient setting, and involved 346 services and 1653 processes. This covered 6 cycles of induction therapy (21 days each, alternating R-CHOP with R-DHAP) [21], apheresis, HDCT followed by stem cell transfusion, posttransplant hospitalization, and continued until AE resolution and patient release, typically occurring 15 days after transplantation, as well as follow-up check-ups until 100 days post-ASCT. While R-CHOP is administered in the outpatient setting, patients are hospitalized for other treatment phases. Out of 49 treatment days, 34 days required inpatient stays, including hospitalization during R-DHAP administration (3 cycles during induction phase), apheresis, and 23 days for the HDCT-ASCT treatment phase (including 15 days post-stem cell transplantation). Lastly, after patient discharge from the hospital, the clinical pathway mapping included 4 outpatient follow-up visits, up to day 99 after the transplantation.

**Fig. 1:**
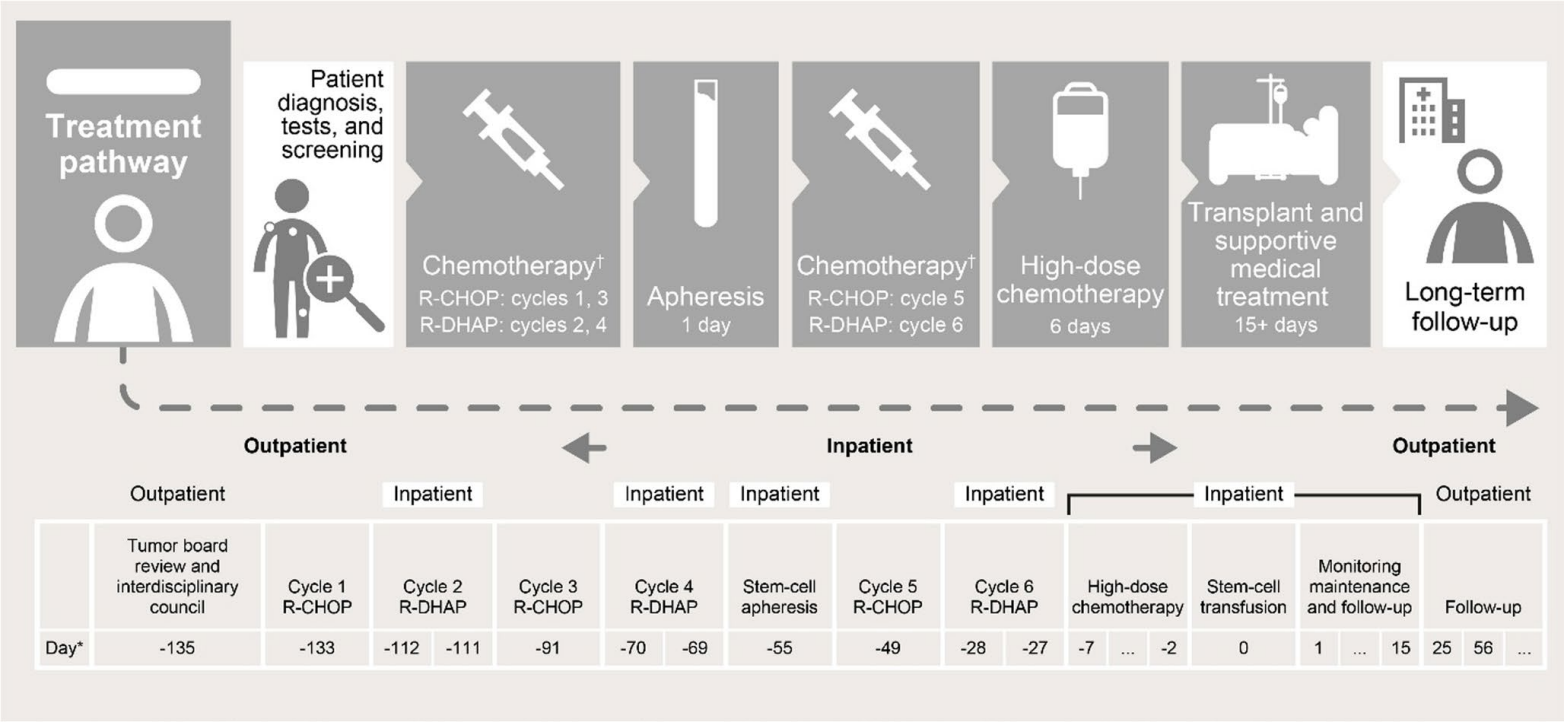
Simplified total clinical pathway. *ASCT, autologous stem cell transplantation; HDCT, high-dose chemotherapy. *Days and routine care between treatment components are not included. †Induction immunochemotherapy consisted of 6 alternating cycles of R-CHOP (rituximab on day 0 or day 1 [i.e., the day prior to or first day of chemotherapy], cyclophosphamide, doxorubicin, and vincristine on day 1, and prednisone on days 1-5), and R-DHAP (rituximab on day 0 or 1,dexamethasone on days 1-4, cytarabine on day 2, and cisplatin on day 1)

The total clinical pathway is displayed in full in Supplementary Fig. S1; a simplified version is illustrated in Fig. 1.

### Healthcare resource utilization

#### Clinical services associated with the total clinical pathway in first-line MCL

The clinical services were classified into 5 categories, as described in Table 1. The duration of each service is presented in Table 1. Inpatient services accounted for most (60%) of the clinical services time, while hospital discharge accounted for the least (4%).

**Table 1:** Description and duration of clinical services associated with the total clinical pathway by category

Category	Details	Duration of clinical services hours: min	Percent of total clinical pathway time
Admission	• Time associated with administrative tasks • Activities encompassing collecting patient histories, planning diagnostic and therapeutic procedures, and ensuring thorough documentation	31:26	10%
Diagnostics	• Comprising a range of laboratory tests and imaging techniques • Includes ordering diagnostic tests, conducting blood draws, and distributing blood samples to various laboratories	42:38	13%
Inpatient services	• Mainly nursing activities and physician responsibilities	189:01	60%
Therapy administration	• Preparation and administration of therapeutic procedures and interventions	40:09	13%
Discharge	• Physician discharge involved performing a final examination, providing discharge summaries, issuing discharge letters, and writing prescriptions • Nursing discharge included giving discharge instructions, documenting follow-up care needs, and educating the patient about recovery	13:51	4%

#### HCRU associated with the total clinical pathway in first-line MCL

The HCP personnel involved with the clinical pathway are doctors, nurses, and functional services personnel (across hematology, cardiology, nuclear medicine, pathology, transfusion medicine, stem cell line, physiotherapy, pulmonology, and radiology departments), as well as psychologists, social services personnel, case management staff, and clinical staff. HCP time associated with both inpatient and outpatient clinical therapy phases of the clinical pathway are described in Supplementary Table S1. The total HCP time across the therapy provision was 317 h.

**Fig. 2:**
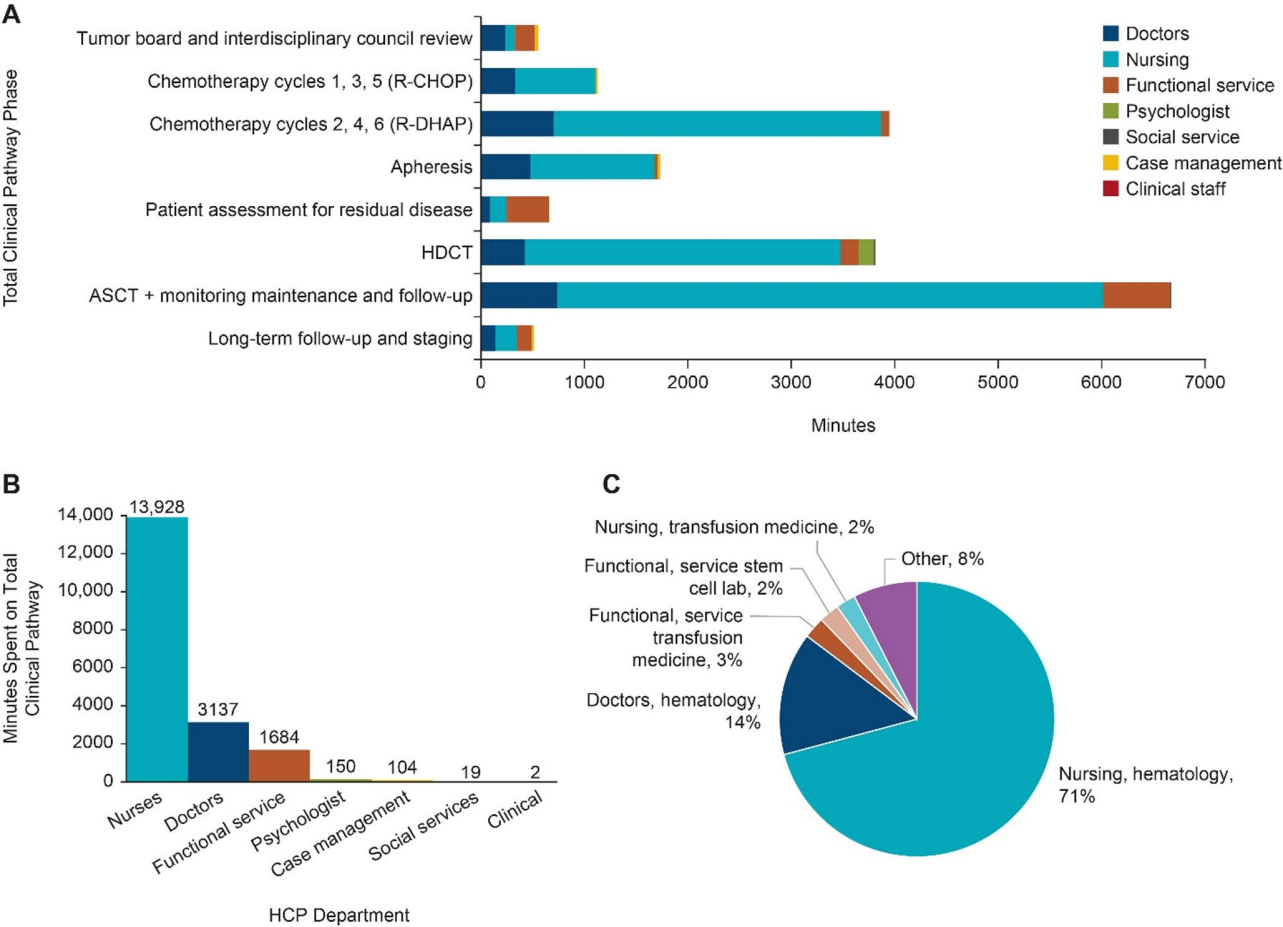
HCP time associated with the total clinical pathway by (**A**) phase and HCP role, (**B**) total time per HCP role, and (**C**) percent of time by HCP role and department. HCP, healthcare professional; R-CHOP, rituximab plus cyclophosphamide, doxorubicin, vincristine, and prednisone; R-DHAP, rituximab plus dexamethasone, cytarabine, cisplatin and prednisone. *Induction immunochemotherapy consisted of 6 alternating cycles of R-CHOP (rituximab on day 0 or day 1 [i.e., the day prior to or first day of chemotherapy], cyclophosphamide, doxorubicin, and vincristine on day 1, and prednisone on days 1-5), and R-DHAP (rituximab on day 0 or 1,dexamethasone on days 1-4, cytarabine on day 2, and cisplatin on day 1)

The most time-intensive procedures in the total clinical pathway were those that occurred during inpatient stays, i.e., during R-DHAP administration, apheresis, and for the HDCT-ASCT treatment phase (Fig. 2A), further details on HCP time per day are shown in Supplementary Fig. S2. Between HCP roles, nurses spent the most time across total clinical pathway procedures (Fig. 2B), with hematology nurses accounting for 71% of total HCP activity (Fig. 2C).

#### HCRU for the HDCT-ASCT phase of the total clinical pathway

The apheresis and HDCT-ASCT phases (consisting of HDCT-ASCT and 15-day inpatient posttransplantation observation) of the total ASCT treatment clinical pathway in first-line MCL involved 31 days of patient-clinician interactions, 25 days of which were in the inpatient setting, 23 days for HDCT-ASCT and 2 days for apheresis. A total of 215 services and 1089 processes were associated with HDCT-ASCT procedures.

The HCP time associated with HDCT-ASCT phases totaled 214 h 42 min, mostly for HCPs in the hematology department (Table 2). This time comprised 24 h for hematologists, 154 h for nursing staff, and 36 h for other functional services (e.g., transfusion medicine and stem cell laboratory) (Table 2). Between HCP roles, nurses spent the most time on HDCT-ASCT phases, with hematology nurses accounting for 72% of all HCP activity (Supplementary Fig. S3).

**Table 2:** Time for patient care and service provision by HCP role

HCP category	Total clinical pathway time* hours: min	HDCT-ASCT time^†^ hours: min	% vs. total procedure
Nursing, hematology	224:38	154:02	69%
Doctors, hematology	45:48	24:01	52%
Functional service, transfusion medicine	8:00	8:00	100%
Nursing, transfusion medicine	7:30	7:30	100%
Functional service, stem cell lab	7:20	7:20	100%
Other	23:49	13:49	58%
Total	317:05	214:42	

*ASCT* indicates autologous stem cell transplantation, *HCP* healthcare professional, *HDCT* high-dose chemotherapy

*HCP time from ASCT-eligibility assessment (day − 135) to 100 days follow-up posttransplantation

^†^HCP time for apheresis, HDCT-ASCT, and 15-day inpatient posttransplantation observation phases of treatment

**Fig. 3:**
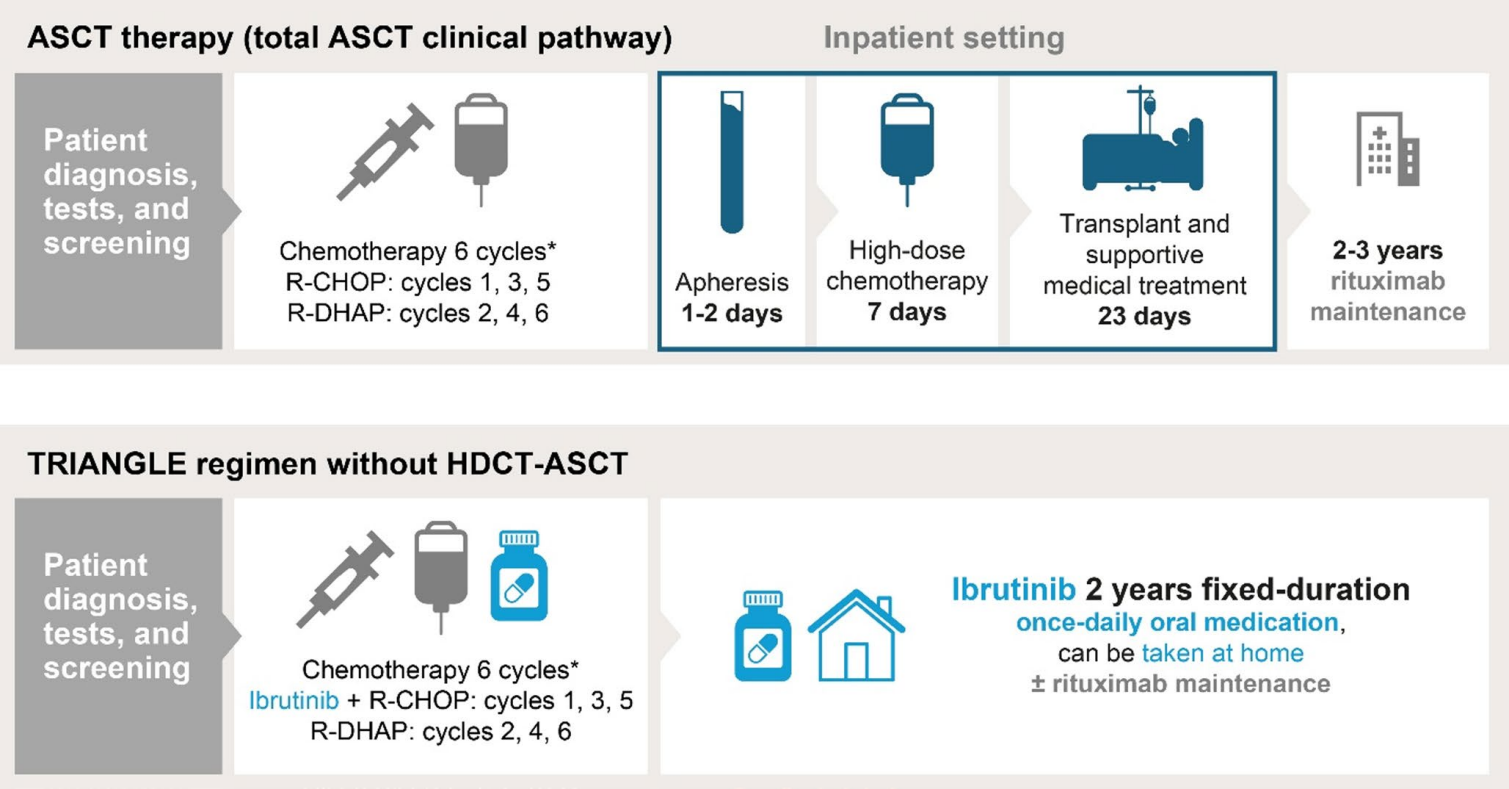
Total ASCT therapy clinical pathway versus TRIANGLE ibrutinib regimen without HDCT-ASCT. ASCT, autologous stem cell transplantation; HDCT, high-dose chemotherapy; R, rituximab; R-CHOP, rituximab plus cyclophosphamide, doxorubicin, vincristine, and prednisone; RDHAP, rituximab plus dexamethasone, cytarabine, cisplatin and prednisone. *Induction immunochemotherapy consisted of 6 alternating cycles of R-CHOP (rituximab on day 0 or day 1 [i.e., the day prior to or first day of chemotherapy], cyclophosphamide, doxorubicin, and vincristine on day 1, and prednisone on days 1-5), and R-DHAP (rituximab on day 0 or 1,dexamethasone on days 1-4, cytarabine on day 2, and cisplatin on day 1).

The simplification of the treatment pathway by removing HDCT-ASCT and replacing with oral medication as per the TRIANGLE ibrutinib regimen without HDCT-ASCT is illustrated in Fig. 3.

### Costs

#### Costs associated with the total ASCT treatment clinical pathway in first-line MCL

**Fig. 4:**
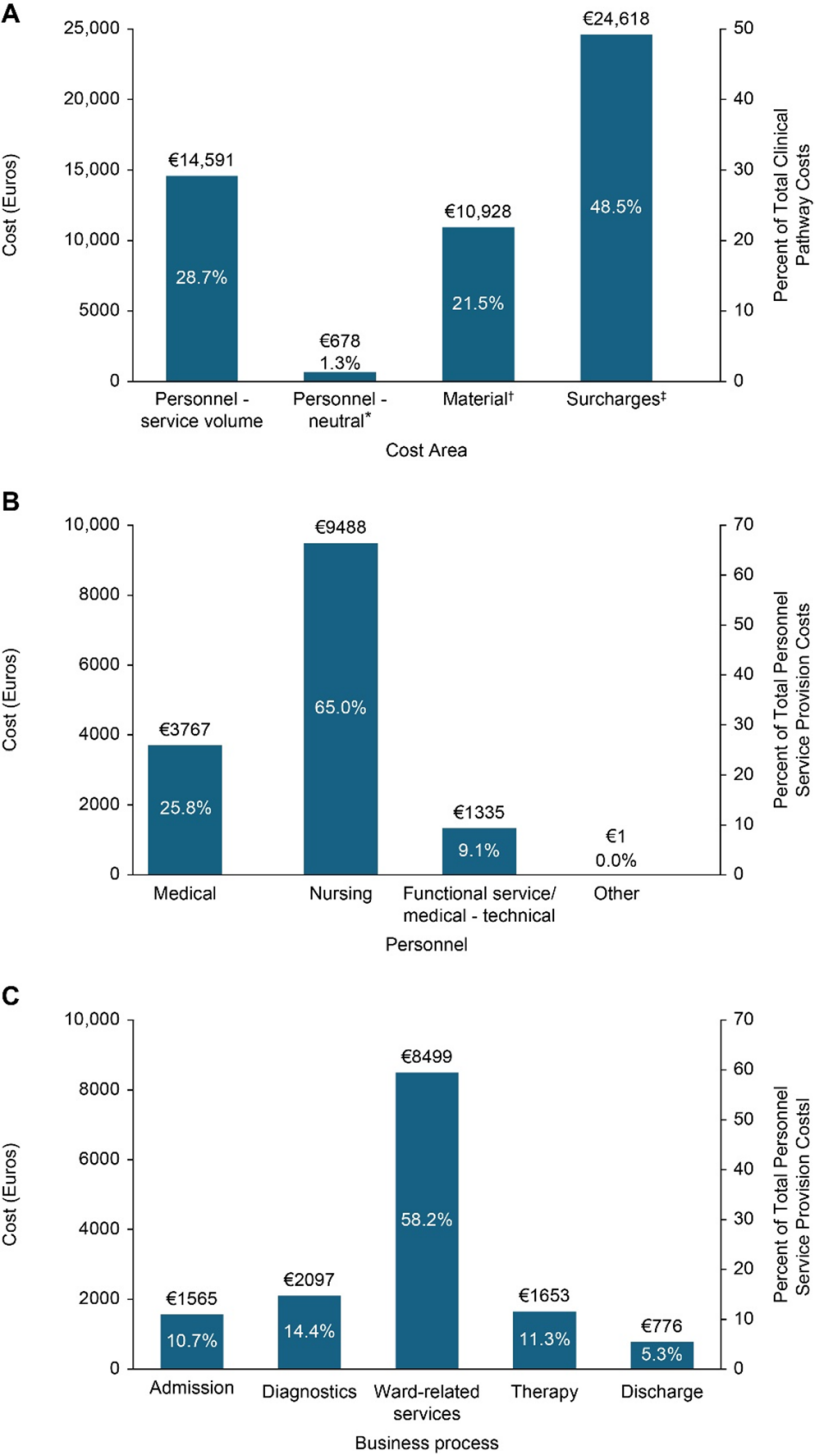
Costs associated with the total clinical pathway by (**A**) cost type, (**B**) personnel role, and (**C**) personnel cost by process type. *Costs incurred for the staff involved, independent of patient volume. †Direct costs related to Zusatzentgelte-relevant medications. ‡Including medical and nonmedical infrastructure, as well as material costs for medical supplies.

The cost analysis, based on the assumption that the modeled clinical pathway represents a complication-free course of treatment, excluding costs associated with management of severe AEs, transfer to ICU, other comorbidities, and long-term complications, showed an associated cost amounting to €50,815. The largest contributor was medical and nonmedical infrastructure and material costs for medical supplies (Fig. 4A). This value is proportionally dependent on various cost drivers such as length of stay or points according to the service catalog. The resource-based time cost calculation emphasizes personnel costs allocated based on exact time required for the execution of a specific process, incurred by clinical staff during the delivery of services, i.e., executing core processes, and accounted for €14,591 (29%) of the total cost (Fig. 4A). Of this, €3767 pertained to medical and €9488 to nursing services (Fig. 4B). The highest personnel costs were observed forward-related services, amounting to €8499 (58% of total service-volume-driven personnel costs) (Fig. 4C).

## Discussion

Until very recently, the only first-line standard of care therapy for younger, fit patients with MCL was HDCT-ASCT, which is associated with acute toxicity and long-term complications for patients and causes considerable demand and cost to healthcare resources. In a national cohort of lymphoma survivors treated with HDCT-ASCT, nearly all experienced moderate or severe late effects at 8 years posttreatment and around half experienced severe or life-threatening late effects, reducing their quality of life [12]. The phase 3 TRIANGLE trial found that the HDCT-ASCT provision of standard therapy could be replaced with an ibrutinib regimen, with clinically beneficial improvements in OS and safety [20, 21], and treatment guidelines now recognize ibrutinib as a first-line treatment option for young, fit patients with MCL [10, 23–27]. This single-center process-orientated analysis aimed to assess the duration of hospitalization, HCRU, and associated costs for healthcare providers of the HDCT-ASCT phase of the clinical pathway for first-line treatment of patients with newly diagnosed MCL who were eligible for ASCT. The study findings showed that resources associated with HDCT-ASCT were considerable. Whilst this study did not establish resource associated with ibrutinib specifically, ibrutinib is an oral treatment that can be taken at home and therefore does not require inpatient hospitalization or procedures, or the costs and resources associated with them. Thus, replacement of HDCT-ASCT with the TRIANGLE ibrutinib regimen would allow assets associated with the procedure to be redirected and alleviate healthcare burden.

In our analysis, the HDCT-ASCT phase of the clinical pathway incurred a minimum of 23 days of hospitalization. Supportive of our findings, a systematic literature review investigating the HCRU of HDCT-ASCT in patients with MCL (including studies from the United States, Switzerland, and Australia) reported a range of 20 to 54 hospitalization days [33]. We found that the HCP time associated with the apheresis and HDCT-ASCT phases, time that could be relinquished if the TRIANGLE ibrutinib regimen without HDCT-ASCT was adopted instead [20, 21], totaled 214 h: 24 h for hematologists, 154 h for nursing staff, and 36 h for other functional services. This highlights a considerable burden on healthcare services, particularly with the current physician and nurse shortages across Europe [34], and removing unnecessary procedures would help to alleviate healthcare demands.

Our study reports direct costs associated with the ASCT total clinical pathway of €50,815. It is important to note that these costs represent only those for a typical hematology ward treatment of a patient with MCL, and do not factor in additional costs that are often encountered for patients with aggressive lymphomas due to complex AEs and ICU transfers, considering mean direct healthcare costs of €107,457 (range, €34,234–328,123) per patient reported in Germany for aggressive lymphomas [19]. Thus, replacing HDCT-ASCT with the TRIANGLE ibrutinib regimen without ASCT could alleviate many of these costs. Given that in the TRIANGLE trial a higher proportion of patients had 3-year remission with ibrutinib without ASCT compared with HDCT-ASCT (87% vs. 76%) [21], salvage therapy is less likely to be needed with ibrutinib, further reducing costs in the long term. Additionally, in TRIANGLE, higher rates of acute toxicity (grade ≥ 3 AEs) were associated with HDCT-ASCT compared with ibrutinib, as well as higher rates of relevant cytopenias (e.g., decreased neutrophil counts: 42% vs. 13%) and sepsis (5% vs. 0%), which pose substantial HCRU and costs due to transfusions and ICU treatment [17, 18, 21]. Of note, secondary malignancies, particularly nonmelanoma skin cancer, myelodysplastic syndrome, and acute myeloid leukemia are relatively frequent complications following HDCT-ASCT, resulting in further direct costs [35, 36]. In addition, revaccination is required following ASCT, increasing HCRU and costs, but would not be anticipated following ibrutinib treatment [37].

Strengths of our analysis include the well-established methodology used and the comprehensive approach, with prior use in over 300 studies. Our findings may be limited by the subjective time estimation methodology, and some degree of uncertainty associated with perceived time spent on tasks. However, this method minimizes time and personnel requirements and, therefore, is particularly useful in clinical practice. This study only investigated HCRU from the perspective of a hospital or staff but does not consider resource use incurred by patients, such as travel time to hospital and payments for arrangements that facilitate time away from home for the ASCT procedure. It should be acknowledged that this study was not designed to conduct a formal cost-effectiveness analysis of treatment alternatives, but rather to quantify the healthcare resource utilization and associated costs attributable to a specific, resource-intensive phase of the clinical pathway — namely, HDCT-ASCT. As such, the costs of ibrutinib or other systemic therapies administered outside of the study pre-specified clinical pathway were beyond the scope of this analysis. A comprehensive understanding of the broader societal implications of replacing HDCT-ASCT with an ibrutinib-containing regimen would necessitate a more holistic assessment, encompassing longer-term downstream resource utilization including those associated with long-term treatment-related complications as well as indirect costs and patient-centered outcomes such as health-related quality of life. The present findings nonetheless provide a valuable, granular characterization of the HCRU associated with HDCT-ASCT and may serve as an important building block to inform such future evaluations.

## Conclusion

In the treatment of newly diagnosed eligible patients with MCL, HDCT-ASCT is associated with at least 23 days of hospitalization, considerable costs, and substantial healthcare resources – assets that could be redirected with the adoption of the oral TRIANGLE ibrutinib regimen without ASCT. 

## Supplementary Information

Below is the link to the electronic supplementary material.

Supplementary Material 1 (PDF 1.03 MB)

## Data Availability

Primary data are available on request from MG: [michael.greiling@iwig-institut.de] .
