# Supplementary material for: Healthcare resource utilization analysis in newly diagnosed mantle cell lymphoma: resource alleviation with adoption of the TRIANGLE ibrutinib regimen without autologous stem cell transplantation
Source: Ann Hematol. 2026 Jul 16;105(8):337. doi: 10.1007/s00277-026-07139-1 (PMC13379447; doi:10.1007/s00277-026-07139-1)
Supplement: Supplementary file 1 — Supplementary Material 1 (PDF 1.03 MB) [file 277_2026_7139_MOESM1_ESM.pdf]

Supplementary Information

Healthcare resource utilization analysis in newly diagnosed mantle cell lymphoma: resource alleviation with adoption of the TRIANGLE ibrutinib regimen without autologous stem cell transplantation

Jonas Wißkirchen[1] • Nora S. Rogmann[1] • Michael Greiling[2] • Frederic Ries[1] • Anke Ohler[1] • Georg Hess[1] • Julia Osygus[3]

[1] Department of Hematology and Medical Oncology, Medical School of the Johannes Gutenberg-University, Mainz, Germany

[2] Institute for Workflow-Management in Health Care (IWiG), European University of Applied Sciences, Cologne, Germany

[3] Institute for Workflow-Management in Health Care (IWiG), Rheine, Germany

Corresponding author: Dr. Jonas Wißkirchen, Department of Hematology and Medical Oncology, Medical School of the Johannes Gutenberg-University, Mainz, Germany

Jonas.Wisskirchen@unimedizin-mainz.de

Fig. S1. Total clinical pathway procedures.

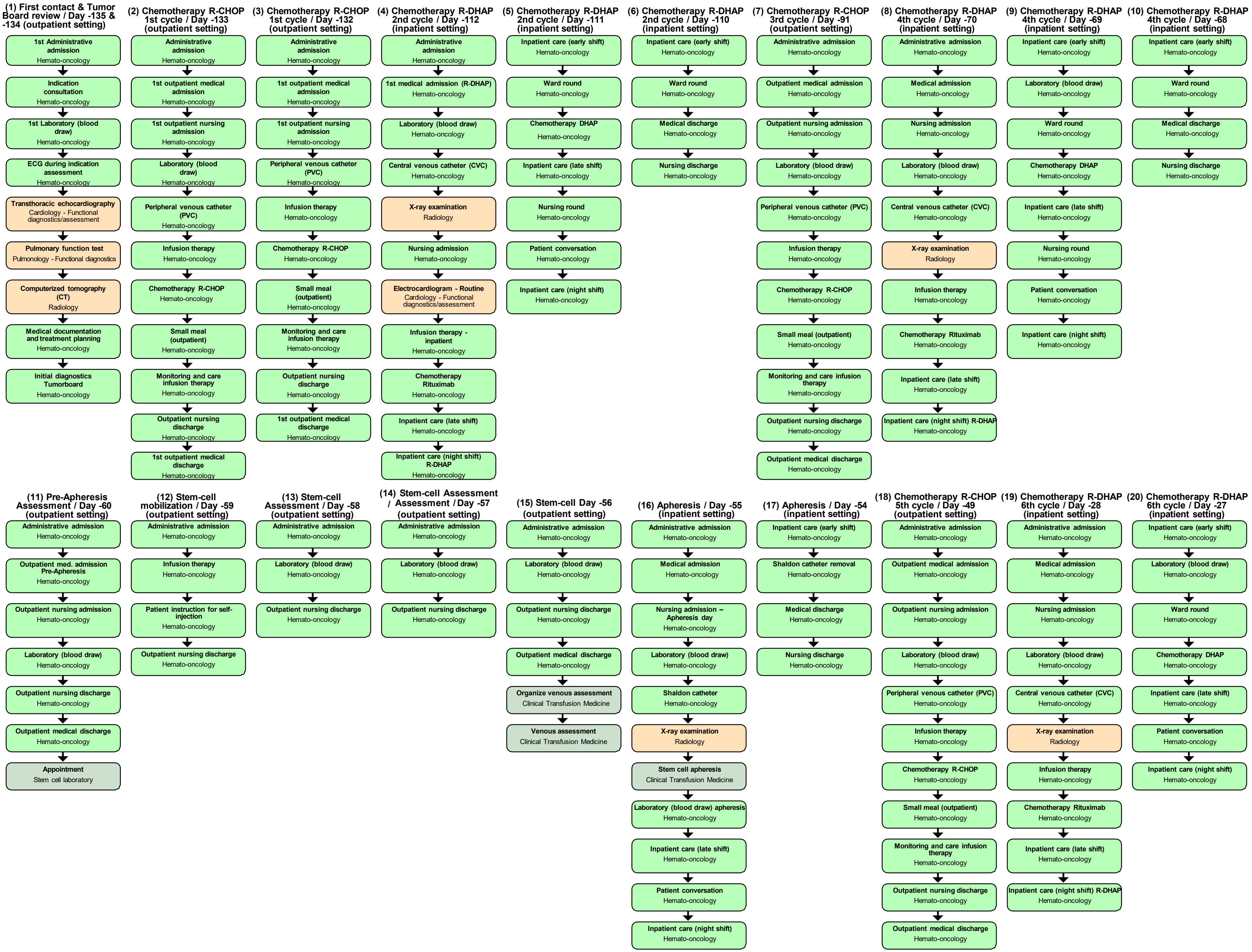

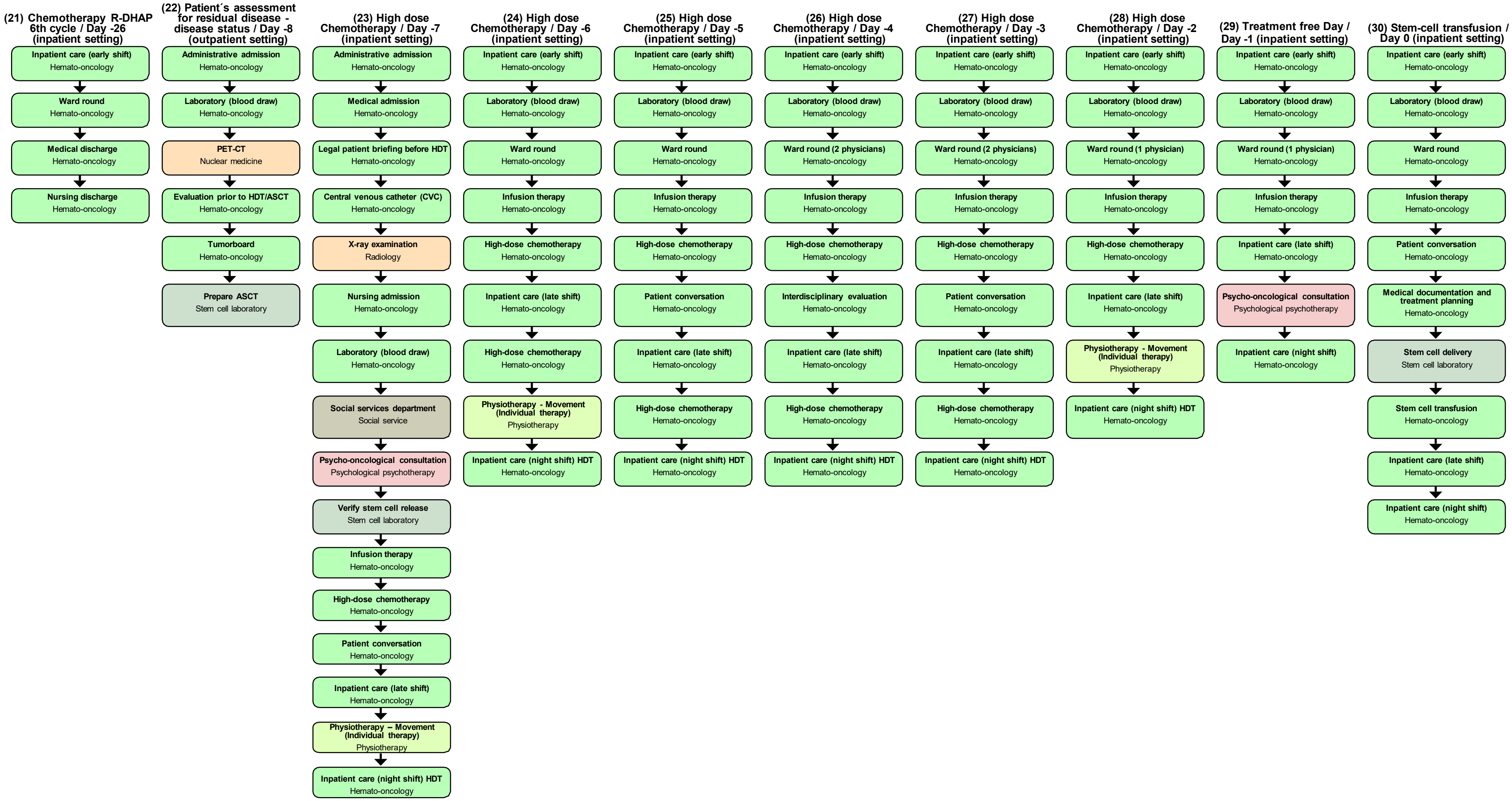
(21) Chemotherapy R-DHAP 6th cycle / Day -26 (inpatient setting)
Inpatient care (early shift) Hemato-oncology
Ward round Hemato-oncology
Medical discharge Hemato-oncology
Nursing discharge Hemato-oncology
(22) Patient´s assessment for residual disease - disease status / Day -8 (outpatient setting)
Administrative admission Hemato-oncology
Laboratory (blood draw) Hemato-oncology
PET-CT Nuclear medicine
Evaluation prior to HDT/ASCT Hemato-oncology
Tumorboard Hemato-oncology
Prepare ASCT Stem cell laboratory
(23) High dose Chemotherapy / Day -7 (inpatient setting)
Administrative admission Hemato-oncology
Medical admission Hemato-oncology
Legal patient briefing before HDT Hemato-oncology
Central venous catheter (CVC) Hemato-oncology
X-ray examination Radiology
Nursing admission Hemato-oncology
Laboratory (blood draw) Hemato-oncology
Social services department Social service
Psycho-oncological consultation Psychological psychotherapy
Verify stem cell release Stem cell laboratory
Infusion therapy Hemato-oncology
High-dose chemotherapy Hemato-oncology
Patient conversation Hemato-oncology
Inpatient care (late shift) Hemato-oncology
Physiotherapy – Movement (Individual therapy) Physiotherapy
Inpatient care (night shift) HDT Hemato-oncology
(24) High dose Chemotherapy / Day -6 (inpatient setting)
Inpatient care (early shift) Hemato-oncology
Laboratory (blood draw) Hemato-oncology
Ward round Hemato-oncology
Infusion therapy Hemato-oncology
High-dose chemotherapy Hemato-oncology
Inpatient care (late shift) Hemato-oncology
High-dose chemotherapy Hemato-oncology
Physiotherapy - Movement (Individual therapy) Physiotherapy
Inpatient care (night shift) HDT Hemato-oncology
(25) High dose Chemotherapy / Day -5 (inpatient setting)
Inpatient care (early shift) Hemato-oncology
Laboratory (blood draw) Hemato-oncology
Ward round Hemato-oncology
Infusion therapy Hemato-oncology
High-dose chemotherapy Hemato-oncology
Patient conversation Hemato-oncology
Inpatient care (late shift) Hemato-oncology
High-dose chemotherapy Hemato-oncology
Inpatient care (night shift) HDT Hemato-oncology
(26) High dose Chemotherapy / Day -4 (inpatient setting)
Inpatient care (early shift) Hemato-oncology
Laboratory (blood draw) Hemato-oncology
Ward round (2 physicians) Hemato-oncology
Infusion therapy Hemato-oncology
High-dose chemotherapy Hemato-oncology
Interdisciplinary evaluation Hemato-oncology
Inpatient care (late shift) Hemato-oncology
High-dose chemotherapy Hemato-oncology
Inpatient care (night shift) HDT Hemato-oncology
(27) High dose Chemotherapy / Day -3 (inpatient setting)
Inpatient care (early shift) Hemato-oncology
Laboratory (blood draw) Hemato-oncology
Ward round (2 physicians) Hemato-oncology
Infusion therapy Hemato-oncology
High-dose chemotherapy Hemato-oncology
Patient conversation Hemato-oncology
Inpatient care (late shift) Hemato-oncology
High-dose chemotherapy Hemato-oncology
Inpatient care (night shift) HDT Hemato-oncology
(28) High dose Chemotherapy / Day -2 (inpatient setting)
Inpatient care (early shift) Hemato-oncology
Laboratory (blood draw) Hemato-oncology
Ward round (1 physician) Hemato-oncology
Infusion therapy Hemato-oncology
High-dose chemotherapy Hemato-oncology
Inpatient care (late shift) Hemato-oncology
Physiotherapy - Movement (Individual therapy) Physiotherapy
Inpatient care (night shift) HDT Hemato-oncology
(29) Treatment free Day / Day -1 (inpatient setting)
Inpatient care (early shift) Hemato-oncology
Laboratory (blood draw) Hemato-oncology
Ward round (1 physician) Hemato-oncology
Infusion therapy Hemato-oncology
Inpatient care (late shift) Hemato-oncology
Psycho-oncological consultation Psychological psychotherapy
Inpatient care (night shift) Hemato-oncology
(30) Stem-cell transfusion / Day 0 (inpatient setting)
Inpatient care (early shift) Hemato-oncology
Laboratory (blood draw) Hemato-oncology
Ward round Hemato-oncology
Infusion therapy Hemato-oncology
Patient conversation Hemato-oncology
Medical documentation and treatment planning Hemato-oncology
Stem cell delivery Stem cell laboratory
Stem cell transfusion Hemato-oncology
Inpatient care (late shift) Hemato-oncology
Inpatient care (night shift) Hemato-oncology

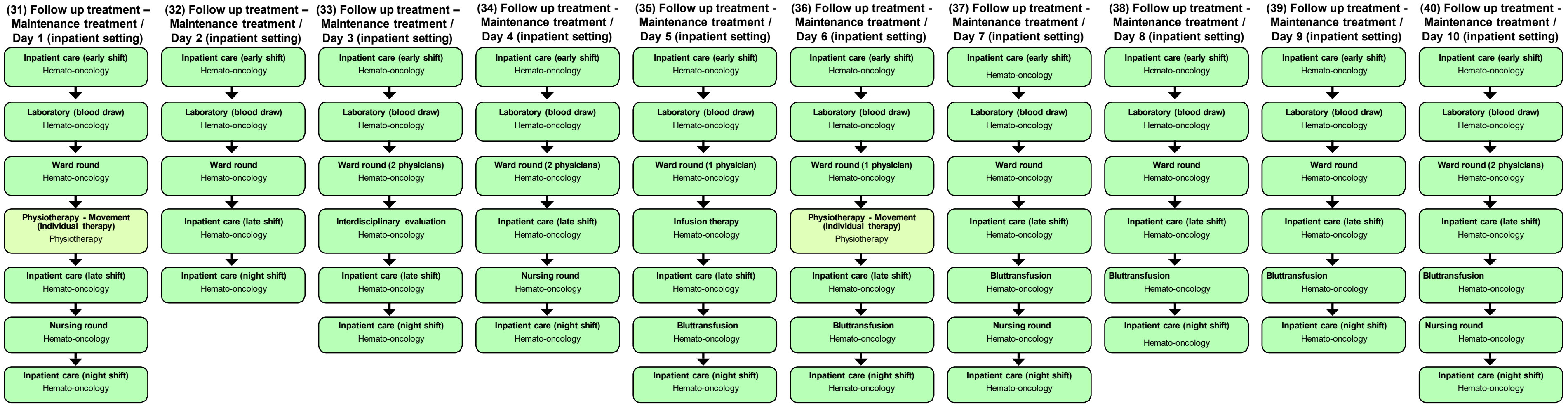
(31) Follow up treatment – Maintenance treatment / Day 1 (inpatient setting)
Inpatient care (early shift) Hemato-oncology
Laboratory (blood draw) Hemato-oncology
Ward round Hemato-oncology
Physiotherapy - Movement (Individual therapy) Physiotherapy
Inpatient care (late shift) Hemato-oncology
Nursing round Hemato-oncology
Inpatient care (night shift) Hemato-oncology
(32) Follow up treatment – Maintenance treatment / Day 2 (inpatient setting)
Inpatient care (early shift) Hemato-oncology
Laboratory (blood draw) Hemato-oncology
Ward round Hemato-oncology
Inpatient care (late shift) Hemato-oncology
Inpatient care (night shift) Hemato-oncology
(33) Follow up treatment – Maintenance treatment / Day 3 (inpatient setting)
Inpatient care (early shift) Hemato-oncology
Laboratory (blood draw) Hemato-oncology
Ward round (2 physicians) Hemato-oncology
Interdisciplinary evaluation Hemato-oncology
Inpatient care (late shift) Hemato-oncology
Inpatient care (night shift) Hemato-oncology
(34) Follow up treatment - Maintenance treatment / Day 4 (inpatient setting)
Inpatient care (early shift) Hemato-oncology
Laboratory (blood draw) Hemato-oncology
Ward round (2 physicians) Hemato-oncology
Inpatient care (late shift) Hemato-oncology
Nursing round Hemato-oncology
Inpatient care (night shift) Hemato-oncology
(35) Follow up treatment - Maintenance treatment / Day 5 (inpatient setting)
Inpatient care (early shift) Hemato-oncology
Laboratory (blood draw) Hemato-oncology
Ward round (1 physician) Hemato-oncology
Infusion therapy Hemato-oncology
Inpatient care (late shift) Hemato-oncology
Bluttransfusion Hemato-oncology
Inpatient care (night shift) Hemato-oncology
(36) Follow up treatment - Maintenance treatment / Day 6 (inpatient setting)
Inpatient care (early shift) Hemato-oncology
Laboratory (blood draw) Hemato-oncology
Ward round (1 physician) Hemato-oncology
Physiotherapy - Movement (Individual therapy) Physiotherapy
Inpatient care (late shift) Hemato-oncology
Bluttransfusion Hemato-oncology
Inpatient care (night shift) Hemato-oncology
(37) Follow up treatment - Maintenance treatment / Day 7 (inpatient setting)
Inpatient care (early shift) Hemato-oncology
Laboratory (blood draw) Hemato-oncology
Ward round Hemato-oncology
Inpatient care (late shift) Hemato-oncology
Bluttransfusion Hemato-oncology
Nursing round Hemato-oncology
Inpatient care (night shift) Hemato-oncology
(38) Follow up treatment - Maintenance treatment / Day 8 (inpatient setting)
Inpatient care (early shift) Hemato-oncology
Laboratory (blood draw) Hemato-oncology
Ward round Hemato-oncology
Inpatient care (late shift) Hemato-oncology
Bluttransfusion Hemato-oncology
Inpatient care (night shift) Hemato-oncology
(39) Follow up treatment - Maintenance treatment / Day 9 (inpatient setting)
Inpatient care (early shift) Hemato-oncology
Laboratory (blood draw) Hemato-oncology
Ward round Hemato-oncology
Inpatient care (late shift) Hemato-oncology
Bluttransfusion Hemato-oncology
Inpatient care (night shift) Hemato-oncology
(40) Follow up treatment - Maintenance treatment / Day 10 (inpatient setting)
Inpatient care (early shift) Hemato-oncology
Laboratory (blood draw) Hemato-oncology
Ward round (2 physicians) Hemato-oncology
Inpatient care (late shift) Hemato-oncology
Bluttransfusion Hemato-oncology
Nursing round Hemato-oncology
Inpatient care (night shift) Hemato-oncology

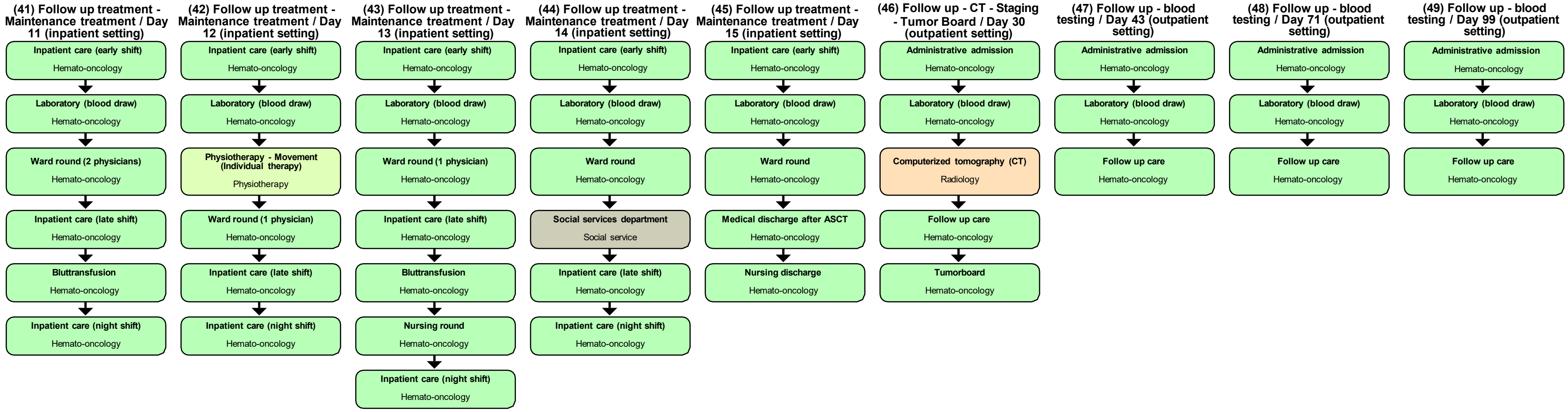

ASCT, autologous stem cell transplantation; HDCT, high-dose chemotherapy.

Fig. S2. Total HCP time utilized per mapped HDCT-ASCT treatment pathway day.

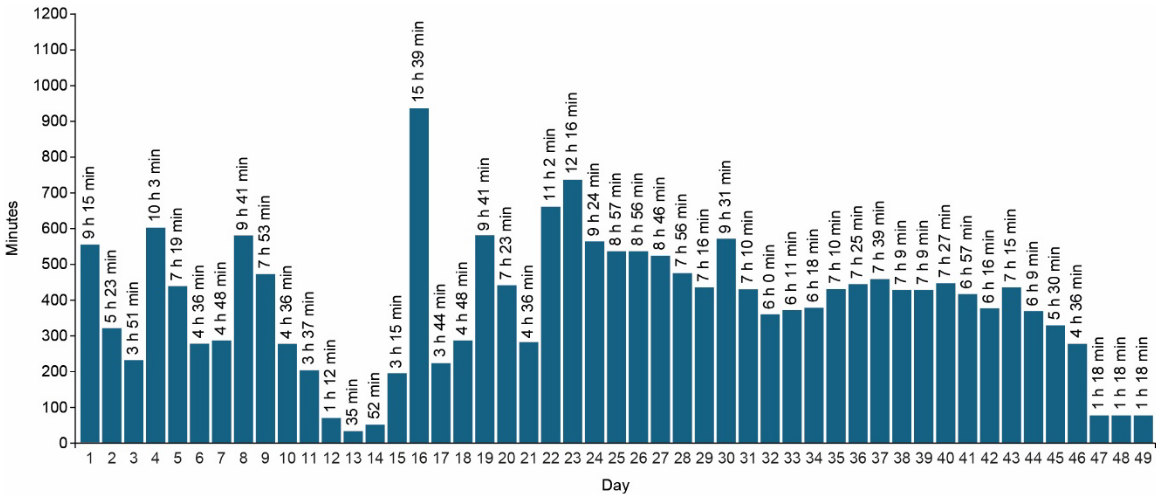

ASCT, autologous stem cell transplantation; HCP, healthcare professional; HDCT, high-dose chemotherapy.

Fig. S3. HCP resources associated with apheresis and HDCT-ASCT.

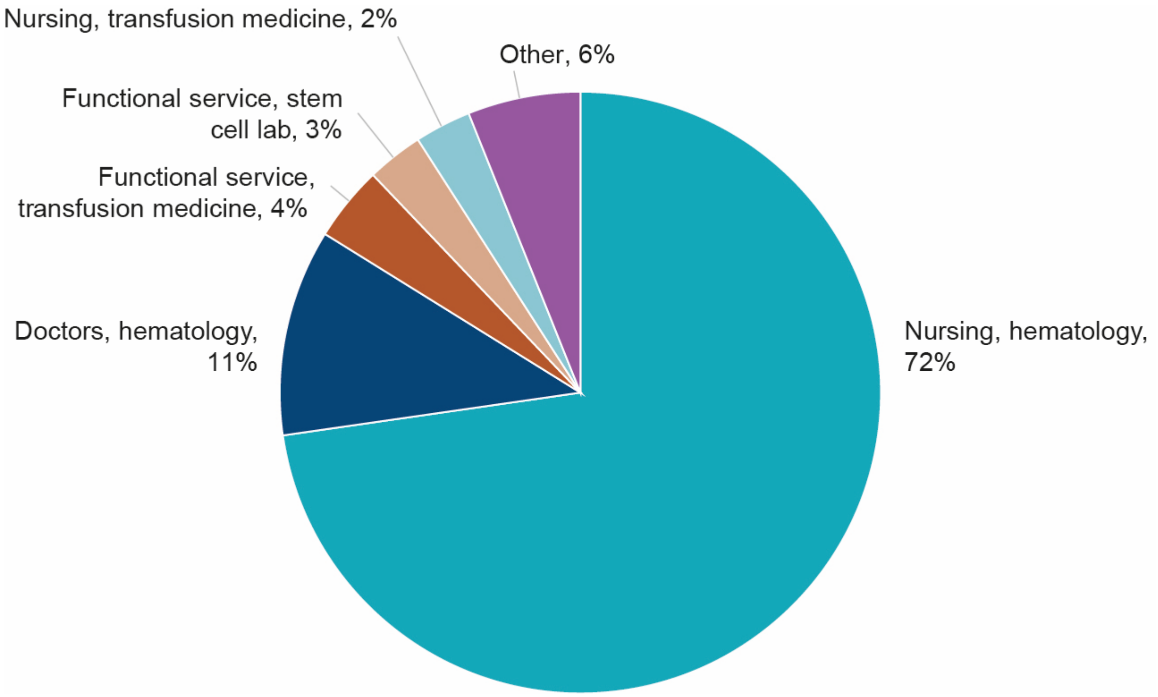

ASCT, autologous stem cell transplantation; HCP, healthcare professional; HDCT, high-dose chemotherapy.

Table S1. HCP time across ASCT therapy phases.

**Clinical pathway**		**Tumor board and interdisciplinary council review**	**Induction chemotherapy cycles 1, 3, 5 R-CHOP***	**Induction chemotherapy cycles 2, 4, 6 R-DHAP***	**Apheresis**	**Residual disease assessment**	**HDCT**	**Stem cell transfusion + monitoring maintenance + follow-up**	**Long-term follow-up + staging**
**Clinical pathway day**		**–135**	**–133, –132, –91, –49**	**–112 to –110, –70 to –68, –28 to –26**	**–60 to –54**	**–8**	**–7 to –1**	**0-15**	**30-99**
Clinical pathway day number		1	2, 3, 7, 18	4-6, 8-10, 19-21	11-17	22	23-29	30-45	46-49
**HCP role/department/time**									
**Doctors**									
Hematology	min	178	333	689	259	26	418	739	107
	**h:min**	**2:58**	**5:33**	**11:29**	**4:19**	**0:26**	**6:58**	**12:19**	**1:47**
Cardiology	min	10	0	0	0	0	0	0	0
	**h:min**	**0:10**	**0:00**	**0:00**	**00:00**	**00:00**	**00:00**	**00:00**	**00:00**
Nuclear medicine	min	7	0	0	0	52	0	0	7
	**h:min**	**0:07**	**0:00**	**0:00**	**0:00**	**0:52**	**0:00**	**0:00**	**0:07**
Pathology	min	7	0	0	0	0	0	0	0
	**h:min**	**0:07**	**0:00**	**0:00**	**00:00**	**00:00**	**00:00**	**00:00**	**00:00**
Pulmonology	min	5	0	0	0	0	0	0	0
	**h:min**	**0:05**	**0:00**	**0:00**	**00:00**	**00:00**	**00:00**	**00:00**	**00:00**
Radiology	min	27	0	15	5	7	5	0	27
	**h:min**	**0:27**	**0:00**	**0:15**	**0:05**	**0:07**	**0:05**	**0:00**	0:27
Transfusion medicine	min	0	0	0	175	0	0	0	0
	**h:min**	**0:00**	**0:00**	**0:00**	**2:55**	**0:00**	**0:00**	**0:00**	**0:00**
Stem cell lab	min	0	0	0	40	0	0	0	0
	**h:min**	**0:00**	**0:00**	**0:00**	**0:40**	**0:00**	**0:00**	**0:00**	**0:00**

**Nurses**									
Hematology	min	91	775	3165	749	162	3055	5277	205
	**h:min**	**1:31**	**12:55**	**52:45**	**12:29**	**2:42**	**50:55**	**87:57**	**3:25**
Transfusion medicine	min	0	0	0	450	0	0	0	0
	**h:min**	**0:00**	**0:00**	**0:00**	**7:30**	**0:00**	**0:00**	**0:00**	**0:00**
**Functional services**									
Transfusion medicine	min	0	0	0	0	0	0	480	0
	**h:min**	**0:00**	**0:00**	**0:00**	**0:00**	**0:00**	**0:00**	**8:00**	**0:00**
Cardiology	min	33	0	10	0	0	0	0	0
	**h:min**	**0:33**	**0:00**	**0:10**	**00:00**	**00:00**	**00:00**	**00:00**	**00:00**
Nuclear medicine	min	0	0	0	0	60	0	0	0
	**h:min**	**0:00**	**0:00**	**0:00**	**0:00**	**1:00**	**0:00**	**0:00**	**0:00**
Physiotherapy	min	0	0	0	0	0	120	120	0
	**h:min**	**0:00**	**0:00**	**0:00**	**0:00**	**0:00**	**2:00**	**2:00**	**0:00**
Pulmonology	min	17	0	0	0	0	0	0	0
	**h:min**	**0:17**	**0:00**	**0:00**	**00:00**	**00:00**	**00:00:00**	**00:00**	**0:00**
Radiology	min	145	0	69	23	0	23	0	145
	**h:min**	**2:25**	**0:00**	**1:09**	**0:23**	**0:00**	**0:23:00**	**0:00**	**2:25**
Stem cell lab	min	0	0	0	9.9	350	30	50	0
	**h:min**	**0:00**	**0:00**	**0:00**	**0:10**	**5:50**	**0:30:00**	**0:50**	**0:00:00**
**Psychologist**									
Psychologist	min	0	0	0	0	0	150	0	0
	**h:min**	**0:00**	**0:00**	**0:00**	**0:00**	**0:00**	**2:30**	**0:00**	**0:00**
**Social services**									
Social services staff	min	0	0	0	0	0	10	10	0
	**h:min**	**0:00**	**0:00**	**0:00**	**0:00**	**0:00**	**0:10**	**0:10**	**0:00**
**Case management**									
Case management staff	min	35	19	0	25	5	0	0	20
	**h:min**	**0:35**	**0:19**	**0:00**	**0:25**	**0:05**	**0:00**	**0:00**	**0:20**

**Clinical**									
Clinical staff	min	0	2	0	0	0	0	0	0
	**h:min**	**0:00**	**0:02**	**0:00**	**00:00**	**00:00**	**00:00**	**00:00**	**00:00**
**Total**									
	min	555	1129	3948	1735	662	3810	6675	511
	**h:min**	**9:15**	**18:49**	**65:48**	**28:55**	**11:02**	**63:30**	**111:15**	**8:30**

ASCT, autologous stem cell transplantation; HCP, healthcare professional; HDCT, high-dose chemotherapy; R-CHOP, rituximab plus cyclophosphamide, doxorubicin, vincristine, and prednisone; R-DHAP, rituximab plus dexamethasone, cytarabine, cisplatin and prednisone.

*Induction immunochemotherapy consisted of 6 alternating cycles of R-CHOP (rituximab on day 0 or day 1 [i.e., the day prior to or first day of chemotherapy], cyclophosphamide, doxorubicin, and vincristine on day 1, and prednisone on days 1-5), and R-DHAP (rituximab on day 0 or 1, dexamethasone on days 1-4, cytarabine on day 2, and cisplatin on day 1).
